# Supplementary material for: NCX-4040, a Unique Nitric Oxide Donor, Induces Reversal of Drug-Resistance in Both ABCB1- and ABCG2-Expressing Multidrug Human Cancer Cells
Source: Cancers (Basel). 2021 Apr 2;13(7):1680. doi: 10.3390/cancers13071680 (PMC8038154; doi:10.3390/cancers13071680)
Supplement: Supplementary file 1 [file cancers-13-01680-s001.pdf]

cancers

Supplementary Materials: NCX-4040, a Unique Nitric Oxide Donor, Induces Reversal of Drug-Resistance in Both ABCB1- and ABCG2-Expressing Multidrug Human Cancer Cells

Birandra K. Sinha, Lalith Perera and Ronald C. Cannon

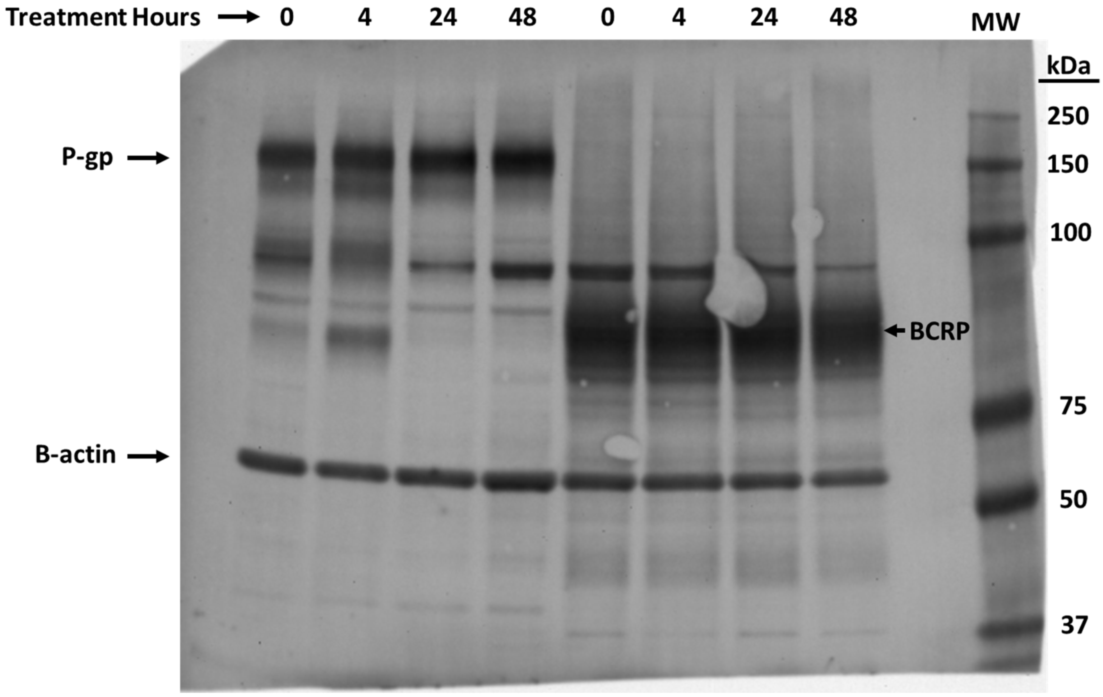

Figure S1. The uncropped Western blots.
